# Applications of artificial intelligence in the field of air pollution: A bibliometric analysis

**DOI:** 10.3389/fpubh.2022.933665

**Published:** 2022-09-07

**Authors:** Qiangqiang Guo, Mengjuan Ren, Shouyuan Wu, Yajia Sun, Jianjian Wang, Qi Wang, Yanfang Ma, Xuping Song, Yaolong Chen

**Affiliations:** ^1^School of Public Health, Lanzhou University, Lanzhou, China; ^2^Department of Health Research Methods, Evidence and Impact, Faculty of Health Sciences, McMaster University, Hamilton, ON, Canada; ^3^McMaster Health Forum, McMaster University, Hamilton, ON, Canada; ^4^School of Chinese Medicine, Hong Kong Baptist University, Kowloon Tong, Hong Kong SAR, China; ^5^Research Unit of Evidence-Based Evaluation and Guidelines, Chinese Academy of Medical Sciences (2021RU017), School of Basic Medical Sciences, Lanzhou University, Lanzhou, China; ^6^Lanzhou University Institute of Health Data Science, Lanzhou, China; ^7^World Health Organization Collaborating Center for Guideline Implementation and Knowledge Translation, Lanzhou, China

**Keywords:** air pollution, public health, artificial intelligence, CiteSpace, bibliometric analysis (BA)

## Abstract

**Background:**

Artificial intelligence (AI) has become widely used in a variety of fields, including disease prediction, environmental monitoring, and pollutant prediction. In recent years, there has also been an increase in the volume of research into the application of AI to air pollution. This study aims to explore the latest trends in the application of AI in the field of air pollution.

**Methods:**

All literature on the application of AI to air pollution was searched from the Web of Science database. CiteSpace 5.8.R1 was used to analyze countries/regions, institutions, authors, keywords and references cited, and to reveal hot spots and frontiers of AI in atmospheric pollution.

**Results:**

Beginning in 1994, publications on AI in air pollution have increased in number, with a surge in research since 2017. The leading country and institution were China (*N* = 524) and the Chinese Academy of Sciences (*N* = 58), followed by the United States (*N* = 455) and Tsinghua University (*N* = 33), respectively. In addition, the United States (0.24) and the England (0.27) showed a high degree of centrality. Most of the identified articles were published in journals related to environmental science; the most cited journal was *Atmospheric Environment*, which reached nearly 1,000 citations. There were few collaborations among authors, institutions and countries. The hot topics were machine learning, air pollution and deep learning. The majority of the researchers concentrated on air pollutant concentration prediction, particularly the combined use of AI and environmental science methods, low-cost air quality sensors, indoor air quality, and thermal comfort.

**Conclusion:**

Researches in the field of AI and air pollution are expanding rapidly in recent years. The majority of scholars are from China and the United States, and the Chinese Academy of Sciences is the dominant research institution. The United States and the England contribute greatly to the development of the cooperation network. Cooperation among research institutions appears to be suboptimal, and strengthening cooperation could greatly benefit this field of research. The prediction of air pollutant concentrations, particularly PM_2.5_, low-cost air quality sensors, and thermal comfort are the current research hotspot.

## Introduction

Air pollution is defined as all destructive effects from any sources which contribute to the pollution of the atmosphere and/or deterioration of the environment (1). It was initially regarded as a threat to respiratory health, however as air pollution research advanced, public health concerns were broadened to include many diseases (2–4), which caused a major threat to human public health. According to the World Health Organization's *COP26 Special Report on Climate Change and Health*, over 90% of people breathe unhealthy levels of outdoor air pollution, which is largely caused by the burning of fossil fuels, which is also driving climate change. It is the first time that air pollution has been listed as a cause of death in the report. Therefore, the World Health Organization launched the “let us breathe clean air” initiative in 2021 (5).

Artificial intelligence (AI) is a new technological science that studies and develops theories, methods, technologies and application systems for simulating, extending and expanding human intelligence (6). One of its main goals is to enable machines to perform complex tasks that would normally require human intelligence. Advantages of AI, such as automatic data extraction, efficiencies in terms of time and labor expenditures, convenience, and long-term sustainability (7, 8), allow for a wide range of applications, including disease prediction, weather forecasting, expert system construction, environmental monitoring, and pollutant prediction (9–14). In recent years, research on the application of AI in air pollution has increased (15). Hu et al. developed a random forest model, incorporating aerosol optical depth data, meteorological fields, and land use variables to estimate daily 24 h averaged ground-level fine particulate matter (PM_2.5_) concentrations over the United States (16). Li et al. developed a novel long short-term memory neural network extended model to predict the air pollutant concentration (17). Huang and Kuo also developed a deep neural network model to apply the PM_2.5_ forecasting system (18).

Bibliometrics is a discipline that uses mathematics, statistics, and other measurement methods to study the distribution structure, quantitative relationships, change patterns, and quantitative management of literature and intelligence. It then investigates specific structures, characteristics, and laws of science and technology, using the literature system and bibliometric characteristics as the object of study. Researchers have begun to use computers for bibliometric work as computers have become more popular. Modern methods and tools, such as computers, are used for data processing and analysis by establishing a systematic and standardized system of data sources and access to primary data. For example, Citation Space (known as “CiteSpace”) is a Java-based information visualization software developed in 2004 by Professor Chaomei Chen of Drexel University's School of Information Science and Technology (USA). It allows the visualization of the structure, patterns and distribution of scientific knowledge. CiteSpace software not only analyzes co-citations in the literature, thus mining the citation space for knowledge clustering and distribution, but also provides the ability to analyze co-occurrence between other knowledge units, allowing for a better presentation and understanding of the progression of scientific knowledge (19). Article co-citation is said to constitute the co-citation relationship when two (or more) articles are simultaneously cited by one or more subsequent articles. When compared to other clusters, an object's silhouette value indicates how similar to its own cluster it is. Modularity and silhouette have values that range from −1 to 1, with values closer to 1 being deemed desirable. An indicator of a node's relevance in a network is called centrality. In their collaborative networks, any research with centrality ratings higher than 0.1 is regarded as influential. Citation bursts, which reveal brief periods of intense scholarly activity, are depicted as red rings. Researchers can identify important hotspots in a field based on these findings.

This study aims to explore the current status of the application of AI in the field of air pollution research using bibliometrics and visual analysis of CiteSpace software (20–22), including the distribution of countries or regions where the research was conducted, authors, and journals, as well as the research hotspots and trends. These analyses then lay the foundation for subsequent relevant research.

## Materials and methods

### Data collection

The data were obtained from the Web of Science (WOS) Core Collection database. Two researchers conducted the search independently on October 13, 2021. The search period was from inception of the database (1980) to October 12, 2021. The main search terms were “air pollution”, “air contamination”, “atmosphere pollutants”, “deep learning”, “machine learning”, and “artificial intelligence” (see the full search strategy in Supplementary material 1). There were no limitations on language, publication year, or record type. Due to the characteristics of CiteSpace, when collecting data, it is not necessary to exclude literature that appears irrelevant, because this may decrease the sensitivity (or accuracy) of searches, resulting in a lost opportunity to discover new associations (23). The categories and impact factor of the journals can be identified by the Journal Citation Reports of the Web of Science database. Two reviewers independently performed literature retrieval and compared results to ensure accuracy and consistency.

### Data analysis

Microsoft Excel 2019 (Redmond WA, USA) was used to conduct data aggregation and analysis. CiteSpace 5.8.R1 software (Drexel University, Philadelphia, PA, USA) was used to analyze the publication characteristics, including record type, publication year, discipline, authors and co-cited authors, countries and institutions, journals, co-cited journals and co-cited references, co-occurrence of keywords and burst keywords. They were identified and described by frequencies. The data was saved in the “Download_ XXX” format and imported into CiteSpace due to the specific data format requirements of that program. The timespan was set as “1990–2021” and slice length was set as 1. The pre-processing of the data mainly removes duplicate documents from the data, and then slices the documents according to time. When words with the same meaning are found in the clustering network, we will add them to the alias list and the alias will be in effect. In addition, the relevant data can also be exported for de-duplication using the Microsoft Excel 2019 (Redmond WA, USA).

## Results

**Figure 1:**
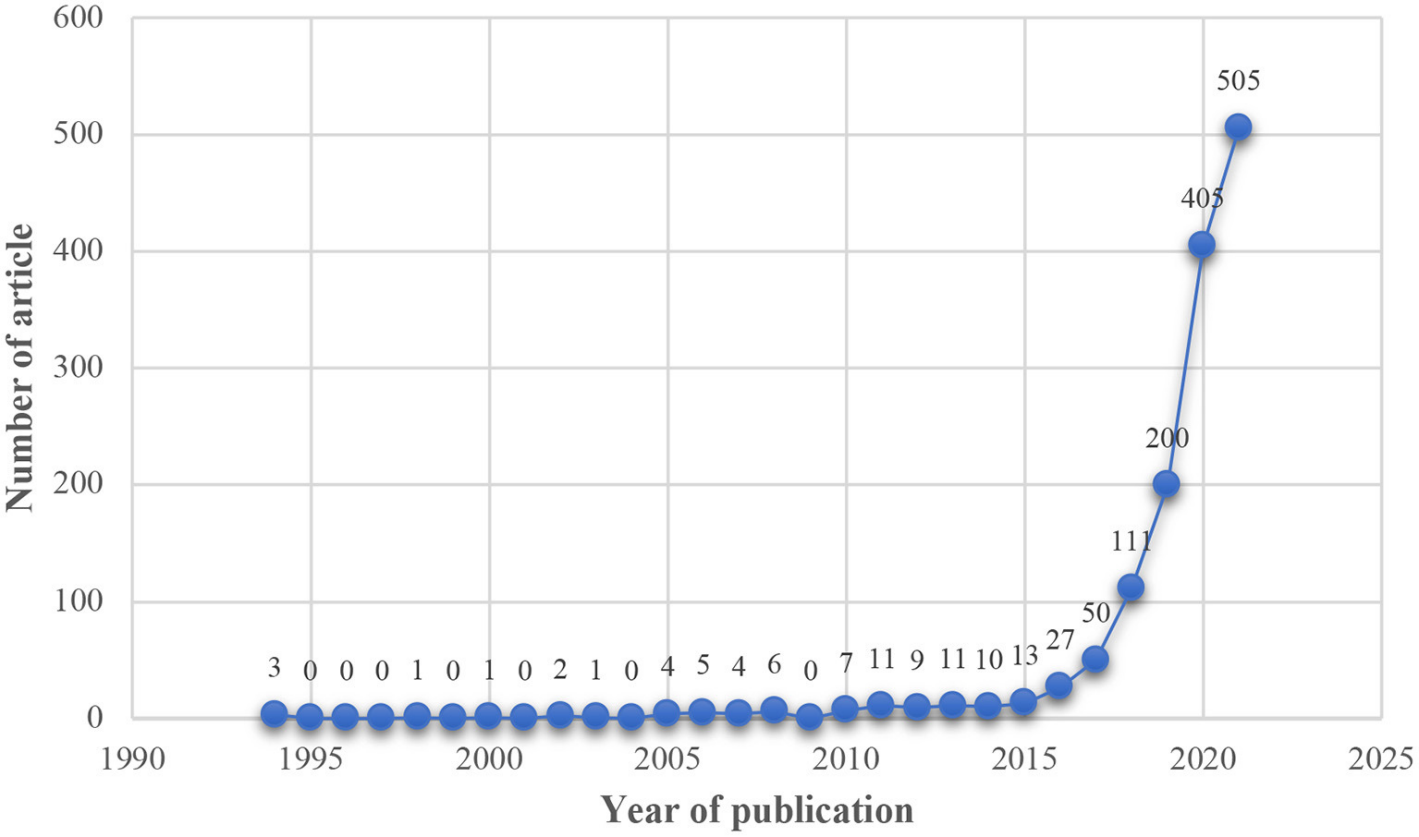
The distribution of the articles by year (*N* = 1,386). The search ended October 12, 2021.

A total of 1,835 records were retrieved in this study, including four publication types. Journal articles were the most frequent type (1,386, 75.5%), followed by proceedings (344, 18.7%), reviews (99, 5.4%), and editorials (6, 0.4%). The first publications appeared in 1994, and since then publications have gradually increased, with a surge starting in 2017 (Figure 1).

### Distribution of countries/regions and institutions

The records came from 93 countries/regions and 466 institutions. The countries with the highest number of publications were China (*N* = 524), followed by the United States (*N* = 455) (Table 1). In addition, the United States (0.24) and the England (0.27) showed a high degree of centrality (Supplementary material 2). The institution with the highest number of publications was the Chinese Academy of Sciences (*N* = 58), and China accounted for eight of the top 10 research institutions (Table 2). Among the top 10 institutions, only Tsinghua University shows a centrality of >0.1, indicating that it is considered important in its collaborative network.

**Table 1:** The top 10 countries/regions publishing research on artificial intelligence and air pollution.

**Rank**	**Countries/regions**	**Count**	**Centrality**
1	China	524	0.08
2	The United States	455	0.24
3	England	136	0.27
4	India	136	0.09
5	South Korea	109	0.04
6	Italy	89	0.14
7	Germany	78	0.05
8	China Taiwan	73	0.06
9	Spain	71	0.12
10	Australia	68	0.19

**Table 2:** The top 10 institutions publishing research on artificial intelligence and air pollution.

**Rank**	**Institutions**	**Count**	**Centrality**
1	Chinese Academy of Sciences (China)	58	0.06
2	Tsinghua University (China)	33	0.12
3	Wuhan University (China)	31	0.01
4	Peking University (China)	29	0.04
5	Nanjing University of Information Science and Technology (China)	27	0.02
6	Zhejiang University (China)	27	0.04
7	Sun Yat Sen University (China)	25	0.04
8	Emory University (USA)	24	0.02
9	University of Chinese Academy of Sciences (China)	23	0.01
10	National Aeronautics and Space Administration (USA)	22	0.07

### Distribution of authors and co-cited authors

In total, all records encompassed 610 authors. The top 10 authors and co-cited authors are shown in Table 3. Liu Y was the most prolific author with 15 articles, followed by Guo YM with 13. Six authors had fewer than 10 publications. The highest co-cited authors were Breiman L (*N* = 284), Hochreiter S (*N* = 155) and Liu Y (*N* = 152).

**Table 3:** The top 10 authors and co-cited authors publishing research on artificial intelligence and air pollution.

**Rank**	**Authors**	**Number**	**Co-cited authors**	**Co-cited number**
1	Liu Y	15	Breiman L	284
2	Guo YM	13	Hochreiter S	155
3	Li SS	11	Liu Y	152
4	Li LF	10	Li X	124
5	Ma J	9	Zhang Y	123
6	Kloog I	9	Di Q	121
7	Fu HB	8	Van Donkelaar A	116
8	Chen GB	8	Hu XF	112
9	Lyapustin A	8	World Health Organization	108
10	Choi Y	7	Lecun Y	107

Liu Y is not only the author with the most published articles, but also the author with the top three citations. He is the Chair of the Gangarosa Department of Environmental Health at the Rollins School of Public Health, Emory University, and a visiting professor at the School of Environment, Tsinghua University. His main research interests include the application of satellite remote sensing in air pollution exposure assessment; the potential impact of climate change on population health; Geographic Information System and spatial statistics. Guo YM, the second most published researcher, Li SS, the third most published researcher, and Chen GB, the eighth most published researcher, are part of a research team. They are from the Department of Epidemiology and Preventive Medicine, School of Public Health and Preventive Medicine at Monash University, where they conduct research on environmental epidemiology, global environmental change, air pollution and health, exposure assessment, remote sensing modeling, and infectious disease modeling.

### Distribution of journals and co-cited journals

The citing journals that published focused on veterinary science, animal science, science, ecology, earth science, and marine biology (see Supplementary material 3). The cited articles focused on systems, computers, environments, toxicology, and nutrition. Of the top 10 co-cited journals, three of these contained articles that have been cited more than 500 times. *Atmospheric Environment* had the highest number of citations (*n* = 958), followed by *Science of the Total Environment* (*n* = 742) (Table 4).

**Table 4:** The top 10 co-cited journals publishing research on artificial intelligence and air pollution.

**Rank**	**Co-cited journals**	**Count**	**Centrality**	**Year**	**IF_2020_**	**Category**
1	Atmospheric Environment	958	0.04	2002	4.798	Meteorology and atmospheric sciences Environmental sciences
2	Science of the Total Environment	742	0.04	2008	7.963	Environmental sciences
3	Environmental Pollution	558	0.03	2008	8.071	Environmental sciences
4	Environmental Science and Technology	460	0.06	1994	9.028	Engineering environmental Environmental sciences
5	Environment International	446	0.05	2008	9.621	Environmental sciences
6	Atmospheric Chemistry and Physics	387	0.03	2014	6.133	Meteorology and atmospheric sciences Environmental sciences
7	Environmental Health Perspectives	370	0.04	2003	9.031	Public, environmental, and occupational health Toxicology Environmental sciences
8	Nature	353	0.03	2001	49.962	Multidisciplinary sciences
9	Environmental Research	345	0.01	2014	6.498	Public, environmental, and occupational health Environmental sciences
10	Machine Learning	339	0.08	2003	2.940	Computer science, artificial intelligence

IF, Impact factor.

### Distribution of keywords

**Figure 2:**
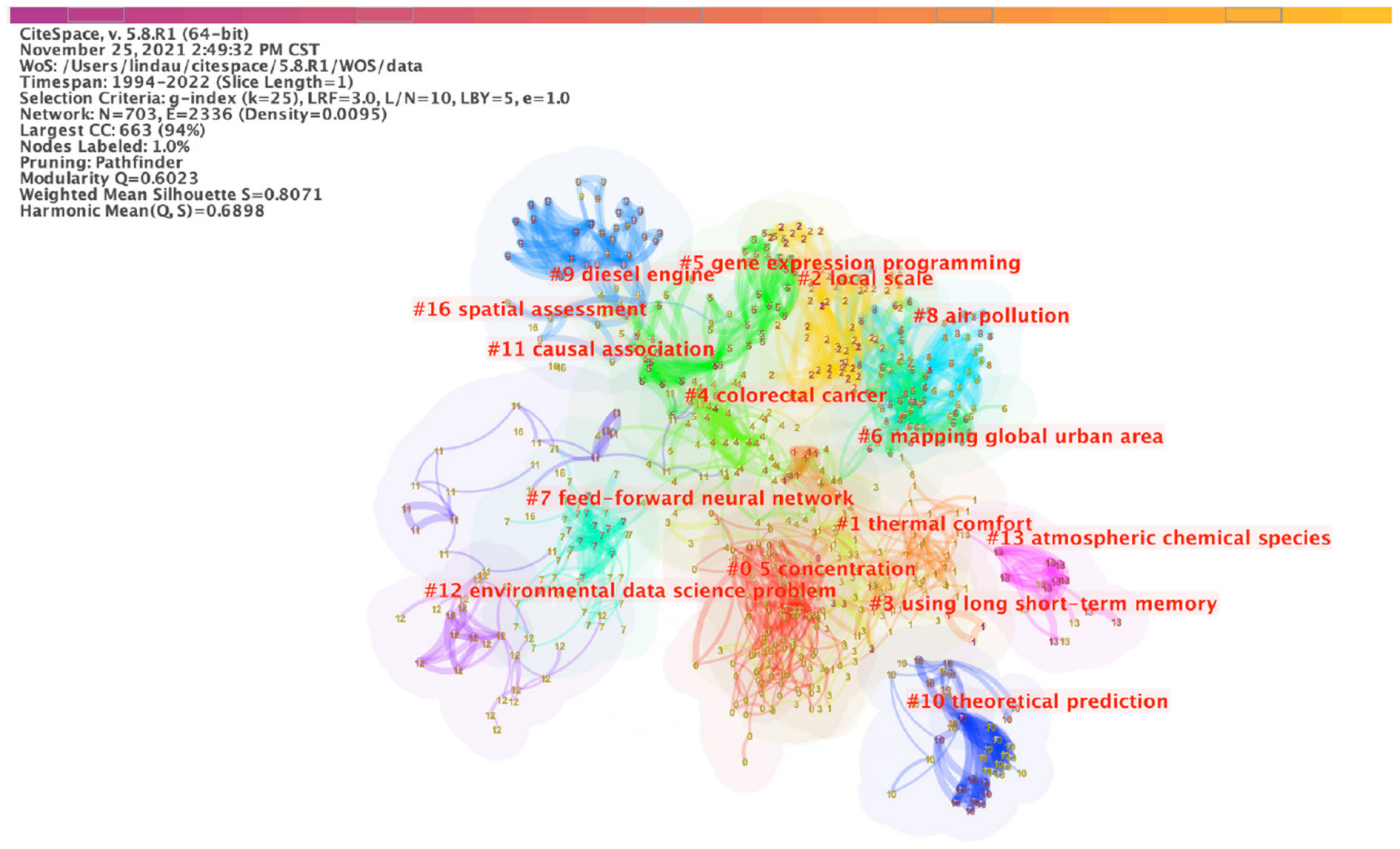
Map of the occurrence of keywords. The nodes in the map represent keywords. The lines between the nodes represent co-occurrence relationships. The larger the node area, the higher the frequency. Each cluster was generated based on the number of keywords under one research domain, not the frequency of keywords.

**Figure 3:**
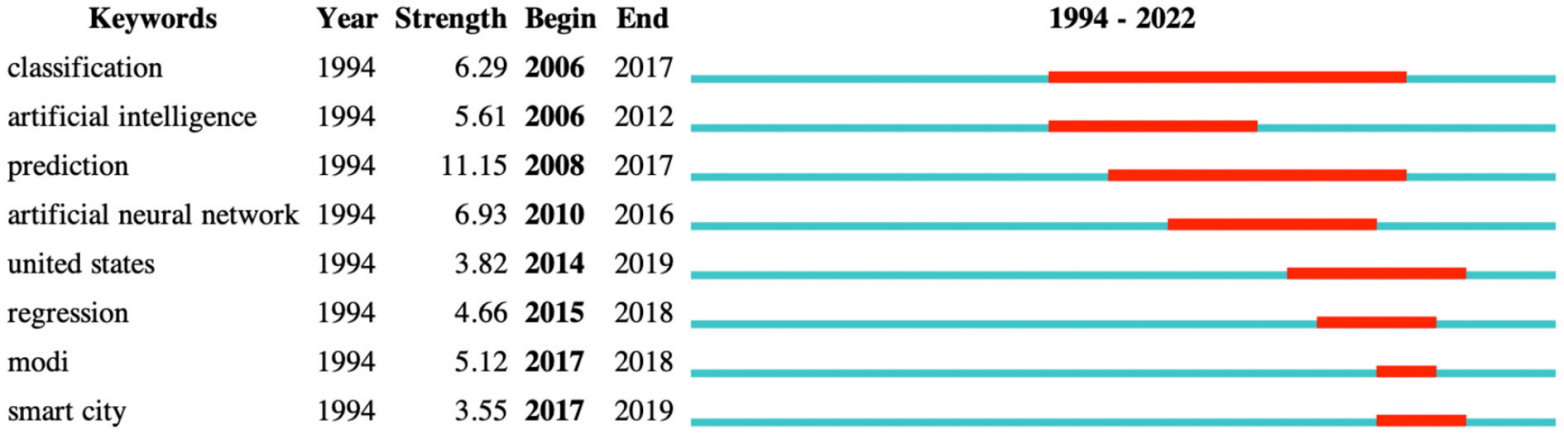
The top eight keywords with the strongest citation bursts. Keywords with a high frequency of citations are represented by red bars, and those with a low frequency by green bars.

The most frequent keywords were machine learning (*N* = 462), air pollution (*N* = 410), and model (*N* = 224) (Supplementary material 4). CiteSpace intelligently classified the research topics into 15 clusters (Figure 2). The modularity (Q) was 0.6023, which was higher than 0.3, indicating that the cluster results were significant. The cluster 0 is the largest cluster (concentration) and cluster 16 is the smallest one (spatial assessment). In recent years, researchers have focused on research about concentration, thermal comfort, air pollution and theoretical prediction (Supplementary material 5). There were eight keywords with the strongest citation bursts, which identifies hot topics (Figure 3). There were no hot topics identified in 2020 or 2021.

### Distribution of references

**Figure 4:**
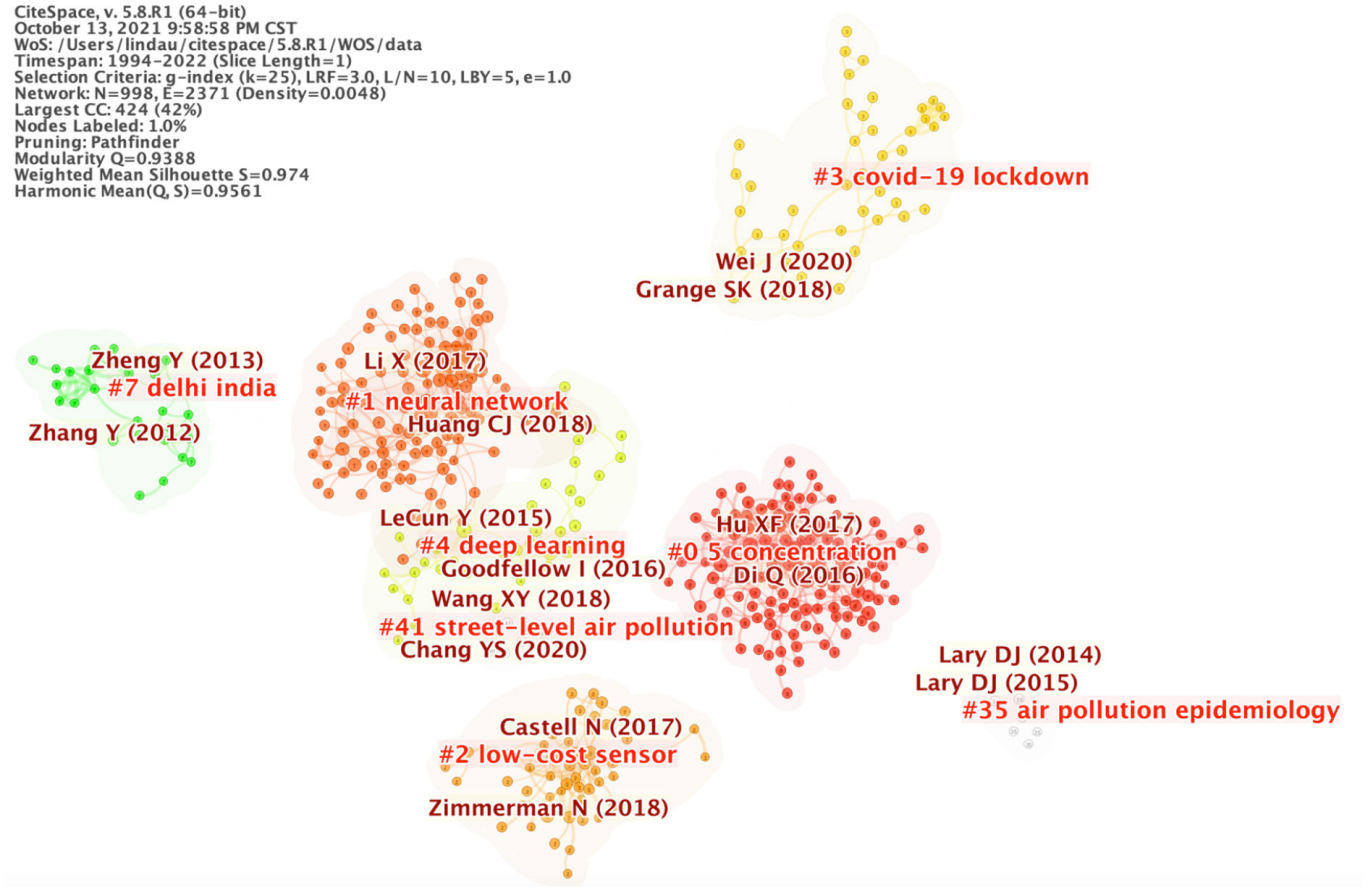
Map of references clustering. The nodes in the map represent references. The lines between the nodes represent co-occurrence relationships. The larger the node area, the higher the frequency.

**Figure 5:**
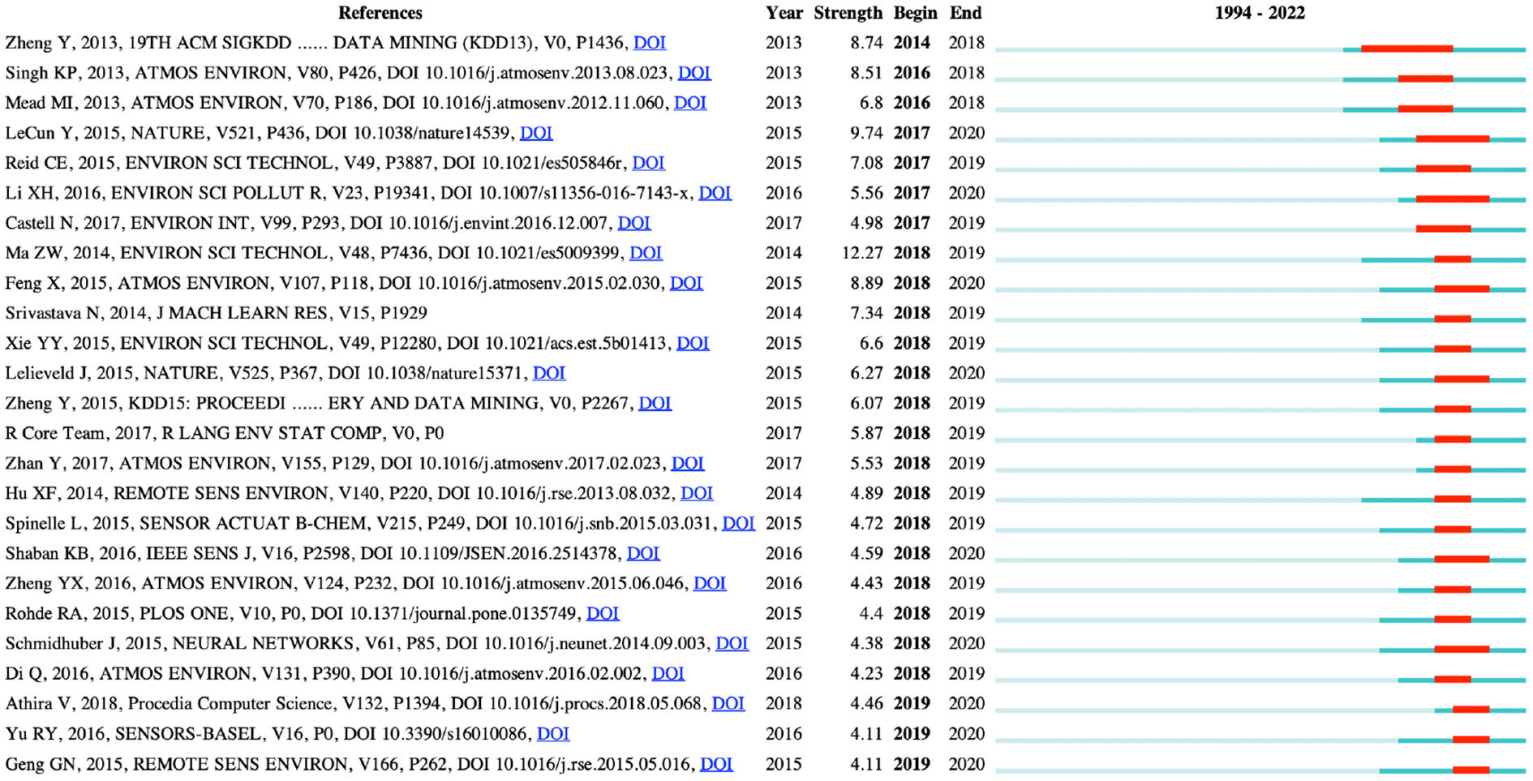
The top 25 references with the strongest citation bursts. Articles with a high frequency of citations are represented by red bars, and those with a low frequency by green bars.

Among the top 10 co-cited references, one article was co-cited more than 90 times. And only one article was co-cited <50 times (Table 5). The map of reference clustering (Figure 4) showed that the collaborative network modularity was 0.9388 and the weighted mean silhouette score 0.974, which are both considered very high (17, 18). Similar to the map of keywords clustering (Figure 2, research on concentrations PM_2.5_, was also the focus for reference clustering (Figure 4). Figure 5 shows the top 25 references with the strongest citation bursts. The first reference with citation bursts appeared in 2014, and the last reference with citation bursts appeared in 2019.

**Table 5:** The Top 10 co-cited references on artificial intelligence and air pollution.

**Rank**	**Title**	**Journal**	**Year**	**First author**	**First author's affiliation**	**Centrality**	**Count**
1	Estimating PM_2.5_ concentrations in the conterminous United States using the random forest approach	Environmental Science and Technology	2017	Hu XF	University of Nevada Reno (USA)	0.01	91
2	Long short-term memory neural network for air pollutant concentration predictions: Method development and evaluation	Environmental Pollution	2017	Li X	Chinese Academy of Sciences (China)	0.03	77
3	A deep CNN-LSTM Model for particulate matter (PM_2.5_) forecasting in smart cities	Sensors	2018	Huang CJ	Jiangxi University of Science and Technology (China)	0.05	63
4	Assessing PM_2.5_ exposures with high spatiotemporal resolution across the continental United States	Environmental Science and Technology	2016	Di Q	Harvard T.H. Chan School of Public Heath (USA)	0.05	62
5	A machine learning method to estimate PM_2.5_ concentrations across China with remote sensing, meteorological and land use information	Science of the Total Environment	2018	Chen GB	Monash University (Australia)	0.00	61
6	Deep learning	Nature	2015	LeCun Y	Facebook AI Research (USA)	0.02	54
7	Estimates and 25-year trends of the global burden of disease attributable to ambient air pollution: an analysis of data from the Global Burden of Diseases Study 2015	Lancet	2017	Cohen AJ	Health Effects Institute (USA)	0.00	54
8	XGBoost: A scalable tree boosting system	KDD16: Proceedings of the 22nd Acm Sigkdd International Conference on Knowledge Discovery and Data Mining	2016	Chen TQ	University of Washington (USA)	0.00	53
9	Spatiotemporal prediction of continuous daily PM_2.5_ concentrations across China using a spatially explicit machine learning algorithm	Atmospheric Environment	2017	Zhan Y	Zhejiang University (China)	0.01	51
10	Artificial neural networks forecasting of PM_2.5_ pollution using air mass trajectory based geographic model and wavelet transformation	Atmospheric Environment	2015	Feng X	Peking University (China)	0.02	49

## Discussion

### Summary of main results

This study explored research trends on the application of AI in the field of air pollution by using bibliometric analysis and data visualization software. The number of studies on the application of AI in the field of air pollution has risen sharply since 2017. Research institutions in China and the United States were the most active in this field, and most studies were published in journals related to environmental science. The research frontier was concentration prediction, especially about PM_2.5_.

### Countries and institutions producing research on artificial intelligence

There may be several reasons for the dominance of Chinese researchers in the field of AI and air pollution. Firstly, on July 8, 2017, China's State Council issued the “New Generation of Artificial Intelligence Development Plan,” which sets out the guiding ideology, strategic targets, key tasks and safeguards for the development of China's new generation of AI in 2030. This Plan highlights “intelligent environmental protection”—namely the establishment of an intelligent monitoring big data platform covering the atmosphere, water, and soil. As well, this plan recommends the development of an intelligent environmental monitoring network and service platform for comprehensive coordination and information sharing. This document also suggests a number of measures, including the development of intelligent prediction models and early warning programs for resource and energy consumption, as well as environmental pollutant emissions. The marked increase in AI-related publications from China since 2017 may reflect the publication of this Plan (24). Secondly, there have been significant advances in science and technology in recent years in China. The *China Internet Development Report (2021)* released by the Internet Society of China reports that the money invested of the AI industry reached 48.0 billion dollars in 2020, an increase of 15% per year since 2020, a rate slightly higher than the global average growth rate (25). Thirdly, Zhu and Liu predicted that China would overtake the United States as the largest publication producer in 2020 or 2021, if all document types were considered (26). Given this, it is likely that the increased number of publications in China will have an impact on publications in a variety of fields.

Although China has a high volume of publications, it has a relatively low centrality of 0.08 compared to the United States (0.24) and the England (0.27). Centrality is an index which measures the importance of a node in a network and is mainly used to measure the value of the bridge function of the node in the entire network structure. Generally, nodes >0.1 are considered relatively important (27). This indicates that, despite the high volume of publications from Chinese research institutions, there are few contacts among them, and their connectivity in the overall connectivity is low. In addition, there does not appear to be close cooperation among Chinese research institutions in this field. More extensive cooperation within China and among countries and research institutions would greatly benefit the field of AI in air pollution.

### Research authors and journals

This study shows that most of the co-cited journals have an environmental science focus: the highest cited journal is *Atmospheric Environment* which reaches nearly 1,000 citations. *Machine Learning* is the only one of the top ten journals which has a singular focus on Computer Science Artificial Intelligence, and the lead researchers mainly belong to the fields of public health and environmental science. This suggests there is a great deal of room for more inter-sectoral research, with input from a broader range of scientists.

### Research keywords

The keywords reflect the themes and core content of the research. The most common keywords could be divided into two categories: artificial intelligence (including machine learning, neural network and deep learning) and air pollution (containing air pollution, air quality, exposure and particular matter). The main research theme was prediction of the concentration of air pollution and model analysis through AI technology. Fifteen clusters were obtained with the mapping of keywords. The nodes on the map of timeline viewer of keywords represent one cluster of research and the denser the nodes are the more researchers pay attention to the cluster. Thermal comfort, which relates to indoor air pollution, is the earliest application and the focus of current research. Thermal comfort refers to the state that most people are satisfied with the objective thermal environment both psychologically and physiologically. Indoor air quality needs to be combined with thermal comfort, where temperature, humidity, and pollutant concentrations are considered in unison and indoor air quality and thermal comfort are inseparable. Initially, traditional mathematical methods were used to give precise definitions of the otherwise vague boundaries between the various levels of thermal sensation. As research progressed, machine learning methods (particularly artificial neural network) were proposed to build algorithmic models for evaluation (28). Artificial neural networks, which are programmed similarly to the human brain, can process large amounts of data. As data is collected, it is processed by various layers in the program, much like neurons in the human brain, and several types of artificial neural networks are available. AI techniques are now also being used to predict indoor thermal comfort. Moreover, the research on the concentration of air pollutants is also a research hotspot. The future research should be focus on the concentration prediction of air pollution.

### Research references

The co-citation can reflect the researchers' attention (22). According to the citation analysis, seven articles predicted the concentration of PM_2.5_ (16–18, 29–32), one study introduced deep learning (33), one analyzed the global burden resulting from environmental air pollution (34), one described a kind of machine learning, namely Tree boosting (35). These authors of high-cited researches mainly come from the United States and China, similar to the countries publishing the most researches in this field.

Deep learning is based on machine learning algorithms, using multiple layers to progressively extract higher-level features from the raw input (36). Learning can be supervised, semi-supervised or unsupervised (37–40). As the map of references clustering showed, the application of deep learning (particularly artificial neural network) in air pollution has attracted much attention (17, 18). It is also the frontier for researchers to develop models for predicting air pollution [including nitrogen dioxide concentration (41), sulfur dioxide concentration (42), ozone density (43), carbon monoxide concentration (44), and so on (45)]. In the past, research hotspots included the study of air pollution in city streets (46–48), epidemiological studies of the temporal and spatial distribution of air pollution (49–51), and low-cost sensors for air quality monitoring (52–54). Since 2020, there has been publication of a large volume of literature on the impact of lockdowns due to Coronavirus Disease 2019 (55–60).

Of the 25 references with the strongest citation bursts, eleven articles focused on using AI methods to predict air pollutant concentrations (31–33, 52, 61–67). In five articles, technologies commonly used in environmental science (e.g., satellite-based aerosol optical depth, satellite remote sensing) were combined with AI models to predict air pollutant concentrations (68–72). Four articles investigated the use of low-cost sensors in air quality monitoring (52, 73–75). One article reviewed deep learning in neural networks (38), another examined the status of air pollution in China (76), and yet another computationally analyzed the major outdoor air pollutants and their sources (77). Srivastava and Salakhutdinov showed one specific artificial intelligence technology (78). Li et al. analyzed the distribution of phthalate esters in water and surface sediments of the Pearl River Estuary (79). It further confirms the research hotspot of predicting air pollutant concentrations. Furthermore, low-cost air quality sensors, particularly mobile air quality sensors, can assist people in recognizing the practical needs of air quality monitoring. Unfortunately, there is a lack of advanced technology to provide an accurate measurement of current conditions. Using big data analysis, advanced algorithms and artificial intelligence techniques, air quality sensors can now provide accurate outdoor air quality analysis and real-time results (80, 81). Lim et al. showed that mobile sampling in conjunction with multiple low-cost air quality monitors could be applied to characterize urban street-level air quality with high spatial resolution, and that machine learning models could further improve model performance (82). Air quality is dynamic and data accuracy can be ensured through calibration, continuous monitoring and air quality data analysis, which is essential to produce valuable and actionable results and recommendations for health decisions in real time.

## Limitations

There are several limitations to this study. Firstly, although we used the WOS for our bibliometric analysis, there are other public and commercially available bibliometric databases, such as Scopus. Due to its long history of construction and the fact that it was the only bibliographic database available before Scopus, the WOS database is currently quite well-known on a global scale. Furthermore, the Scopus database covers fewer articles published before 1996, which has some limitations. And only the WOS database was taken into consideration as the data source for this study because the research institution where this research team is based has only purchased access to it. Secondly, the keywords used in the search strategy (e.g., particular matter, sulfur dioxide, and nitrogen dioxide, etc.) related to specific contaminants in the air, which may not lead to the identification of studies in all aspects of air pollution. Thirdly, some top-ranked keywords were uninformative in isolation (e.g., model, pollution, and exposure) and thus could not be analyzed. Therefore, future studies could employ broader search strategies to further explore this literature.

## Conclusion

Researches in the field of AI and air pollution are expanding rapidly in recent years. The majority of scholars are from China and the United States. But the United States and the England contribute greatly to the development of the cooperation network. The Chinese Academy of Sciences is the dominant research institution. Cooperation among research institutions appears to be suboptimal, and strengthening cooperation could greatly benefit this field of research. The prediction of air pollutant concentrations, particularly combined use of AI and environmental science method, low-cost air quality sensors, indoor air quality, and thermal comfort are the current research hotspot.

## Data availability statement

The original contributions presented in the study are included in the article/Supplementary material, further inquiries can be directed to the corresponding author/s.

## Author contributions

QG, XS, and YC designed the study. QG collected the data, analyzed the data and wrote the original draft. MR collected the data and reviewed the manuscript. QG, SW, YS, JW, QW, YM, XS, and YC contributed to modifying and reviewing. All authors have read and approved the manuscript.

## Funding

This study was supported by the Fundamental Research Funds for the Central Universities (Grant No. lzujbky-2021-ey13) and the Science and Technology Program of Gansu Province (Grant No. 20JR5RA262). The funders were not involved in the study design, collection, analysis, interpretation of data, the writing of this article, or the decision to submit it for publication.

## Conflict of interest

The authors declare that the research was conducted in the absence of any commercial or financial relationships that could be construed as a potential conflict of interest.

## Publisher's note

All claims expressed in this article are solely those of the authors and do not necessarily represent those of their affiliated organizations, or those of the publisher, the editors and the reviewers. Any product that may be evaluated in this article, or claim that may be made by its manufacturer, is not guaranteed or endorsed by the publisher.
