# Supplementary material for: Applications of artificial intelligence in the field of air pollution: A bibliometric analysis
Source: Front Public Health. 2022 Sep 7;10:933665. doi: 10.3389/fpubh.2022.933665 (PMC9490423; doi:10.3389/fpubh.2022.933665)
Supplement: Supplementary file 1 [file Data_Sheet_1.pdf]

Supplementary material 1. Search strategy

6	**(#5) AND #2**	Edit | Add to Search	1,835
5	**((#1) OR #3) OR #4**	Edit | Add to Search	569,450
4	**"air pollutions"** (Topic) or **"air pollutants"** (Topic) or **"air contaminations"** (Topic) or **"atmosphere pollutions"** (Topic) or **"atmosphere pollutants"** (Topic) or **"atmosphere contaminations"** (Topic) or **"atmospheric pollutions"** (Topic) or **"atmospheric pollutants"** (Topic) or **"atmospheric contaminations"** (Topic)	Edit | Add to Search	20,969
3	**"particulate matter"** (Topic) or **"inhalable particle"** (Topic) or **"fine particle"** (Topic) or **"particle matter"** (Topic) or **"thoracic particles"** (Topic) or **"carbon monoxide"** (Topic) or **"nitrogen dioxide"** (Topic) or **"sulphur dioxide"** (Topic) or **"sulfurous anhydride"** (Topic) or **"sulfur dioxide"** (Topic) or **"nitric oxide"** (Topic)	Edit | Add to Search	474,000
2	**"artificial intelligence"** (Topic) or **"machine learning"** (Topic) or **"Deep learning"** (Topic)	Edit | Add to Search	328,362
1	**"air pollution"** (Topic) or **"air pollutant"** (Topic) or **"air polluted"** (Topic) or **"air quality"** (Topic) or **"air contamination"** (Topic) or **"atmosphere pollution"** (Topic) or **"atmosphere pollutant"** (Topic) or **"atmosphere contamination"** (Topic) or **"atmospheric pollution"** (Topic) or **"atmospheric pollutant"** (Topic) or **"atmospheric contamination"** (Topic)	Edit | Add to Search	130,739
