# Supplementary material for: Applications of artificial intelligence in the field of air pollution: A bibliometric analysis
Source: Front Public Health. 2022 Sep 7;10:933665. doi: 10.3389/fpubh.2022.933665 (PMC9490423; doi:10.3389/fpubh.2022.933665)
Supplement: Supplementary file 2 [file Data_Sheet_2.pdf]

Supplementary material 2. Distribution of publications from different countries/regions

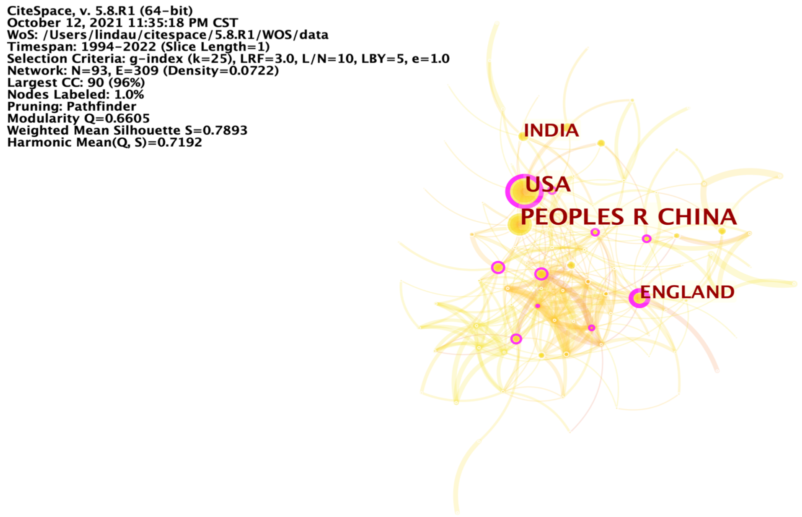

This figure shows the distribution of publications from different countries or regions. The font size of the country name represents the number of articles published by the country/region. The purple ring of the circles indicates the core country/region. The thickness of the curved connecting lines represents the collaborative intensity between the countries/regions.
