# Supplementary figures and images for: Applications of artificial intelligence in the field of air pollution: A bibliometric analysis
Source: Front Public Health. 2022 Sep 7;10:933665. doi: 10.3389/fpubh.2022.933665 (PMC9490423; doi:10.3389/fpubh.2022.933665)

Supplementary material 3. Dual-map overlays of discipline and journal analysis

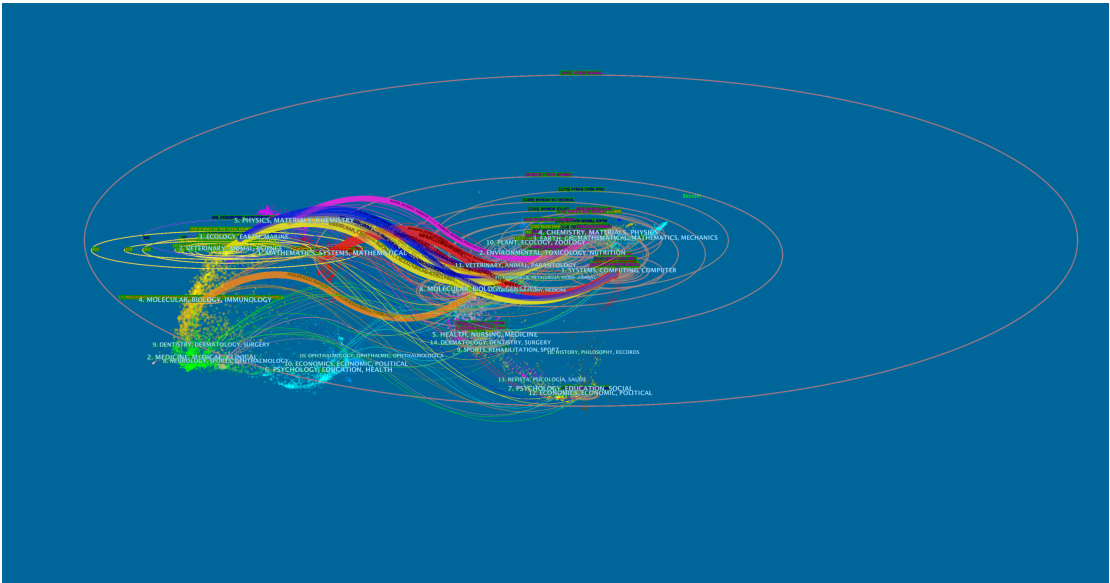

Supplement: Supplementary file 3 [file Data_Sheet_3.pdf]
