# Supplementary material for: Applications of artificial intelligence in the field of air pollution: A bibliometric analysis
Source: Front Public Health. 2022 Sep 7;10:933665. doi: 10.3389/fpubh.2022.933665 (PMC9490423; doi:10.3389/fpubh.2022.933665)
Supplement: Supplementary file 4 [file Data_Sheet_4.pdf]

Supplementary material 4. The top 10 keywords publishing research on artificial intelligence and air pollution

Rank	Keywords	Count	Centrality	Year
1	machine learning	462	0.14	2002
2	air pollution	410	0.18	2004
3	model	224	0.10	2004
4	neural network	223	0.14	1998
5	air quality	223	0.06	2008
6	deep learning	209	0.01	2016
7	prediction	199	0.05	2003
8	particulate matter	196	0.03	2003
9	pollution	177	0.02	2008
10	exposure	133	0.02	2014
