# Supplementary material for: Applications of artificial intelligence in the field of air pollution: A bibliometric analysis
Source: Front Public Health. 2022 Sep 7;10:933665. doi: 10.3389/fpubh.2022.933665 (PMC9490423; doi:10.3389/fpubh.2022.933665)
Supplement: Supplementary file 5 [file Data_Sheet_5.pdf]

Supplementary material 5. Map of timeline viewer of keywords

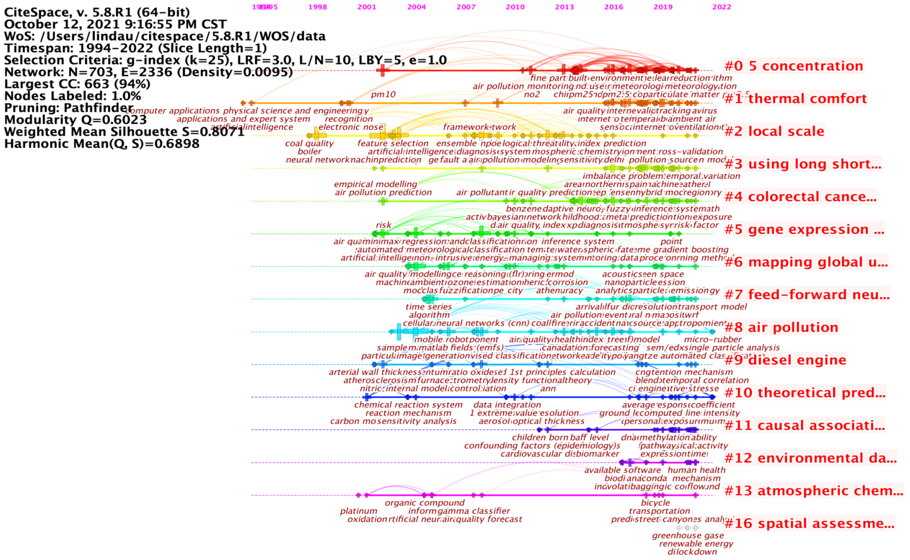

Note: #0 5 concentration, #1 thermal comfort, #2 local scale, #3 using long short-term memory, #4 colorectal cancer, #5 gene expression programming, #6 mapping global urban area, #7 feed-forward neural network, #8 air pollution, #9 diesel engine, #10 theoretical prediction, #11 causal association, #12 environmental data science problem, #13 atmospheric chemical species, #16 spatial assessment.
